# Discontinuity Predictions of Porosity and Hydraulic Conductivity Based on Electrical Resistivity in Slopes through Deep Learning Algorithms

**DOI:** 10.3390/s21041412

**Published:** 2021-02-18

**Authors:** Seung-Jae Lee, Hyung-Koo Yoon

**Affiliations:** Department of Construction and Disaster Prevention Engineering, Daejeon University, Daejeon 34520, Korea; seungjae1215@edu.dju.ac.kr

**Keywords:** deep learning algorithm, electrical resistivity, field test, hydraulic conductivity, hyperparameter, porosity

## Abstract

Electrical resistivity is used to obtain various types of information for soil strata. Hence, the prediction of electrical resistivity is helpful to predict the future behavior of soil. The objective of this study is to apply deep learning algorithms, including deep neural network (DNN), long-short term memory (LSTM), and gated recurrent unit (GRU), to determine the reliability of electrical resistivity predictions to find the discontinuity of porosity and hydraulic conductivity. New DNN-based algorithms, i.e., LSTM-DNN and GRU-DNN, are also applied in this study. The electrical resistivity values are obtained using 101 electrodes installed at 2 m intervals on a mountaintop, and a Wenner array is selected to simplify the electrode installation and measurement. A total of 1650 electrical resistivity values are obtained for one measurement considering the electrode spacing, and accumulated data measured for 15 months are used in the deep learning analysis. A constant ratio of 6:2:2 among the training, validation, and test data, respectively, is used for the measured electrical resistivity, and the hyperparameters in each algorithm are moderated to improve the reliability. Based on the deep learning model results, the distributions of porosity and hydraulic conductivity are deduced, and an average depth of 25 m is estimated for the discontinuity depth. This paper shows that the deep learning technique is well used to predict electrical resistivity, porosity, hydraulic conductivity, and discontinuity depth.

## 1. Introduction

Among the various geophysical survey methods, the electrical resistivity method has been used in various fields to analyze the behavior of the soil subsurface. In particular, electrical resistivity has been theoretically correlated with porosity, hydraulic conductivity, and water content, which are fundamental properties in geotechnical engineering [1,2]. Thus, efforts have been made to determine slope behavior through electrical resistivity. Lebourg et al. [3] and Jomard et al. [4] evaluated slope stability by considering an increase in water content and rock weathering using the electrical resistivity method. Perrone et al. [5] also used the electrical resistivity method to predict the water content of landslides and perform a risk assessment with additionally measured horizontal displacement. Electrical resistivity is an alternative method for evaluating the stability of slopes.

Localized discontinuities of hydraulic conductivity appear as various particles carried on the outside and inside the slope in this deposited layer. Hydraulic conductivity is related to the pore water pressure [6]; thus, if the fine content is deposited below the coarse-grained content, the pore water pressure may rise above the fine content layer due to relatively low hydraulic conductivity [7]. The different hydraulic conductivities in even one region have a great influence on soil water infiltration from matric suction [8]. Thus, the observation of hydraulic conductivity alternation is important to understand the mechanism of failure in slopes because pore water pressure and infiltration are highly related to landslides. Hydraulic conductivity refers to the velocity of the fluid flow through the porous medium, and electrical resistivity denotes the resistance of the current flow in the porous medium since the soil particles are a non-conductive material. Thus, it is possible to predict hydraulic conductivity under both saturated and unsaturated conditions from electrical resistivity because they characterize the same area [9,10].

Deep learning algorithms, which are part of the fourth-era industrial technology, are used to predict the behavior of land slopes. Gorsevski et al. [11] predicted the possibility of slope failure by linking artificial neural network techniques to LiDAR results. Six factors that affect the possibility of landslides were used as the input data: the slope, solar radiation, catchment, plan curvature, profile curvature, and wetness index. Further, the sigmoid function was used as the activation function. Yang et al. [12] predicted the probability of landslides by combining displacement data with the support vector machine (SVM) and long-short term memory (LSTM) deep learning algorithms. Both sigmoid and hyperbolic tangent (tanh) were used as the activation function. Huang et al. [13] proposed a fully connected spare autoencoder to predict landslide susceptibility, comparing it with results based on a support vector machine and a backpropagation neural network. Liang et al. [14] also used various deep learning techniques, including gradient boosting decision tree, random forest, and k-means clustering for susceptibility mapping, and the result based on the gradient boosting decision tree is the most reliable. Deep learning techniques have been used to predict subsurface characterization; however, there are limitations when selecting a suitable algorithm, activation function, or hyperparameter because the selection of these parameters depends on the data characteristics. Therefore, in this study, the most reliable algorithm and hyperparameter for predicting the electrical resistivity for each selected algorithm are proposed.

In this study, among the various deep learning algorithms available, we selected the deep neural network (DNN), which is a suitable technique for regression analysis, long-short term memory (LSTM), and gated recurrent unit (GRU), which are frequently used in time-series data analysis, as the target algorithms. DNN-based complex LSTM-DNN and GUR-DNN techniques were also used to combine the merits of regression analysis and time-series computations. This paper begins with a theoretical background of electrical resistivity and deep learning algorithms, and the field for measuring electrical resistivity is also described in detail. The measured electrical resistivity was verified by responding to rainfall and temperature alternation, and the reliability of the determined hyperparameter was also evaluated. The deep learning algorithms that yielded the best prediction results were selected under this experimental condition, and the most reasonable hyperparameters for each algorithm are proposed. Finally, the discontinuity depth based on the bilinear method through profiles of porosity and hydraulic conductivity converted from electrical resistivity are discussed.

## 2. Background Theory

The relationship among electrical resistivity, porosity and hydraulic conductivity, and deep learning, which were used for analyzing the characteristics of soil and predicting future soil behavior, are described as follows.

### 2.1. The Relationship between Electrical Resistivity and Hydraulic Conductivity

Electrical resistivity is the degree of disturbance of the electric flow, and electrical resistivity is inversely related to electrical conductivity, which indicates how well electricity passes through a medium. Electrical resistivity can be expressed using the electrical resistance (R), cross-sectional area (S), and current path (L), as expressed in Equation (1). However, it is difficult to calculate the correct area and path. Thus, the apparent electrical resistivity is calculated using the geometric coefficient (K) with the current (I) and voltage difference (ΔV), as expressed in Equation (1). In the electrical resistivity method, four electrodes are usually used to inject current and measure the voltage. Various array methods are available, depending on electrode spacing [15]. The Wenner array, in which the electrodes are equally spaced, was adopted in this study considering its ease of installation and measurement efficiency. The geometric coefficient (K) of the Wenner array is expressed as K = 2πa, with ‘a’ being the distance between two successive electrodes, as indicated in Equation (1).

(1) $$\rho =R\left(\frac{S}{L}\right)\approx K\left(\frac{\Delta V}{I}\right)\approx 2\pi a\left(\frac{\Delta V}{I}\right)$$

where the units of ρ, R, S, L, V, I are Ω·m, Ω, m^2^, m, V, and A, respectively.

Archie [16] suggested the intrinsic formation factor (FF_i_) through fluid electrical resistivity (ρ_W_) and bulk electrical resistivity (ρ_O_) under 100% saturated conditions with the same fluid. The FF_i_ is related to porosity (φ) with the tortuosity factor (α) and cementation factor (m) shown in Equation (2). The α and m values are determined by the properties of the soil particles, and the FF can be rearranged with the bulk electrical resistivity (ρ), saturation (S), and saturation constant (n). The saturation constant can be obtained from additional experiments based on a semi-log scale.

(2) $${FF}_{i}=\frac{\rho_{O}}{\rho_{W}}=\alpha \cdot \varphi^{-m}=\frac{\rho }{\rho_{W}}\cdot S^{n}$$

Equation (2) is often used in the field of geotechnical and geological engineering to derive porosity as a design parameter [17,18]. In addition, studies to understand particle properties were performed by linking the contact characteristics and cementation effects of soil materials since α and m values were related to particle shape [19,20]. There is an alternative method to find porosity through dynamic cone penetration index (DCPI) estimated by dynamic cone penetrometer as follows:

(3) $$e=0.43+0.0027\frac{{\mathrm{DCPI}}_{300}}{D_{50}}$$

where e is the void ratio. DCPI_300_ denotes the DCPI at a penetration depth of 300 mm, and D_50_ is the average diameter of the particle. The porosity (φ) can be obtained from the void ratio (e) with the relationship of φ =e/1+e [21].

In a porous medium, electricity mainly travels through the porosity when the particle is a non-conductor. The hydraulic conductivity expresses how fast the fluid moves in the porous medium, and the flow path of the fluid is mainly porous, similar to the movement of electrical resistivity. Thus, Lesmes and Friedman [10] suggested the relationship between the apparent formation factor (FF_a_) and hydraulic conductivity (K_S_) of unsaturated soil through the Kozeny–Carmen equation as follows.

(4) $$K_{S}=\frac{10^{-5}}{{FF}_{a}\cdot {\left(S_{p}\right)}^{C}}$$

where S_P_ denotes the specific surface area per unit volume, which can be deduced from the ratio between the shape factor (SF) and effective diameter (D_eff_). The ranges of SF are generally 6.0 to 6.6 for round particles and 7.7 to 8.4 for angular media. The effective diameter can be calculated through a sieve analysis. c is a constant with a range of 2.8 to 4.6 depending on the material type [22], and it is related to the saturation [23] FF_a_ indicates the ratio of bulk electrical resistivity to fluid electrical resistivity. FF_a_ and FF_i_ are the same for a clay-free medium [24]. 

### 2.2. Deep Learning Algorithm Theory

Deep learning algorithms are used in various fields to recognize the patterns of objects after continuous learning and predict the future behavior of objects. In this study, DNN, which is widely used as an algorithm in various fields, was selected. Additionally, LSTM and GRU, based on the recurrent neural network (RNN), were also utilized because they are specialized for predicting characteristics that change with time. Furthermore, complex algorithms were constructed by combining LSTM-DNN and GRU-DNN to improve the reliability of the predicted data.

#### 2.2.1. Deep Neural Network (DNN)

DNN was developed in 1943 [25] for extracting trends from observed data and predicting the future behavior of objects through regression analysis. DNN has been used in several fields because of its relative simplicity. DNN is known as a feedforward neural network because it cannot reflect the recurrent measured value. However, DNN can be applied to short-term time series [26]. Thus, DNN was applied in this study. DNN is generally composed of an input layer, a hidden layer combined with three or more nodes, and an output layer [25]; the nonlinear function is applied to the hidden layer. Each node consists of input data (X), weight matrices (w), and bias vectors (b). The relationship between the input data (X) and the final output data (O_DNN_) can be expressed using Equation (5) with a calculation function (σ).

(5) $$O_{\mathrm{DNN}}=\sigma \left(\underset{n}{\sum }X\cdot W_{n}+b_{n}\right)$$

#### 2.2.2. Long-Short Term Memory (LSTM)

LSTM, derived from RNN, can be used to connect the previous and current measured data as a time series and is thus used for predictions considering a time-lapse. In the case of RNN, if numerous past data exist, the cumulative value, which is calculated using tanh of the activation function, creates a vanishing gradient problem that quickly converges to zero when applying the backpropagation method [27]. In other words, the presence of more data increases the difficulty of reflecting previous data. The LSTM was proposed in 1997 to compensate for the weakness of the RNN [28]. The memory cells configured in the horizontal direction cause a time-lapse effect. Each LSTM cell consists of a forget gate (F), input gate (I), cell update gate (C), and output gate (O). When the input data (X) and hidden vector sequence (H) are defined as expressed in Equation (6), all the gates are arranged as a vector convolution, as presented in Equation (7):

X = {x_1_, x_2_, x_3_,… x_n_} H = {h_1_, h_2_, h_3_,… h_n_} (6)

Forget gate: F_t_ = σ (w_f_ · [h_t−1_, x_t_] + b_f_) at time t Input gate: F_i_ = σ (w_i_ · [h_t−1_, x_i_] + b_i_) at time t Cell update gate: C_t_ = F_t_ · C_t−1_ + (σ (w_i_ · [h_t−1_, x_t_] + b_i_)) · (tanh (w_c_ · [h_t−1_, x_c_] + b_i_) at time t Output gate: O_t_ = tanh (C_t_) · σ (w_o_ · [h_t−1_, x_t_] + b_o_) at time t (7)

where σ, w, and b denote the activation functions, weight matrices, and bias vectors, respectively, as in the DNN. LSTM mainly uses sigmoid functions as an activation function. The subscripts f, i, and o indicate values corresponding to the forget gate, input gate, and output gate, respectively [28].

Each memory cell of the LSTM is an output to the cell update gate (C_t_) and output gate (H_t_), and Ct represents how much weighting is used in the next step by storing the previous data using the F_t_ function. In addition, O_t_ determines the validity of the output data and provides information reflected in the next memory cell.

#### 2.2.3. Gated Recurrent Unit (GRU)

Similar to LSTM, GRU, which was developed in 2014 [29], is also derived from RNN. GRU is an alternative algorithm used for solving the three complicated gates of LSTM, and only two reset gates (R) and update gates (U) are used for predicting the time-elapsed data as a lightweight algorithm. The GRU architecture maintains a memory cell format similar to that of LSTM because the GRU architecture is based on RNN [29]. If the input data (X) and hidden vector sequence (H) are the same as those in Equation (6), the reset gate (R) and update gate (U) are expressed as shown in Equation (8).

Reset gate: R_t_ = σ (w_r_ · [h_t−1_, x_t_] + b_r_) at time t Update gate: U_t_ = σ (w_u_ · [h_t−1_, x_t_] + b_u_) at time t (8)

Therefore, the final output gate (O) is summarized as presented in Equation (9). The algorithm is constructed without the cell update gate (C) of the LSTM. If the Ut value is 1, this value is reflected in the current value, but if it is 0, then the corresponding memory cell is passed. Equation (9) shows that Ut can be used to determine whether the current value is reflected.

Output gate: O_t_ = (1−U_t_) · h_t−1_ + U_t_ · (tanh (w_t_ · [R_t_ · h_t−1_, x_t_])) at time t (9)

The symbols in Equations (8) and (9) have the same meaning as those in Equation (7).

#### 2.2.4. Coupled Algorithms Based on Deep Neural Network (DNN) with Long-Short Term Memory (LSTM) and Gated Recurrent Unit (GRU)

DNN, LSTM, and GRU can be used to predict nonlinear data and trends. DNN is specialized for feature extraction, whereas LSTM and GRU are mainly used for the trend analysis of time-series data. Therefore, different dimensions are used to achieve the purposes of different algorithms. DNN is a two-dimensional array (size x feature), and both LSTM and GRU have a three-dimensional array (size x feature x time step). Although the aforementioned deep learning algorithms have different input arrays, similar to DNN, both LSTM and GRU can be used to derive the output dimension as a two-dimensional array after reflecting the time step in the calculation. Therefore, it is possible to link each algorithm. In this study, the LSTM-DNN and GRU-DNN complex algorithms were constructed and applied by connecting DNN to the LSTM and GRU algorithms to improve the reliability of the predicted data, thereby exhibiting the advantages of each deep learning algorithm.

#### 2.2.5. Performance Evaluation

The root mean square error (RMSE), mean absolute error (MAE), and coefficient of determination (R^2^) were used to evaluate the reliability of each algorithm, as presented in Equations (10)–(12), respectively. While the RMSE shows the difference between the measured and predicted electrical resistivity values, both pairs of continuous variables are used in MAE for calculating the performance [30]. R^2^ represents the proportion of the variance.

(10) $$\mathrm{RMSE}=\sqrt{\frac{1}{n}\sum_{t=1}^{n}{\left(Z(s_{t})-(Ž(s_{t})\right)}^{2}}$$

(11) $$\mathrm{MAE}=\frac{1}{n}\sum_{t=1}^{n}\left|Z(s_{t})-Ž(s_{t})\right|$$

(12) $$R^{2}=1-\frac{\sum_{t=1}^{n}{\left(Z(s_{t})-Ž(s_{t})\right)}^{2}}{\sum_{t=1}^{n}{\left(Z(s_{t})-\dot{Z}\right)}^{2}}$$

where Z(s_t_), Ž(s_t_), and Ż denote the measured electrical resistivity, the predicted electrical resistivity, and the average value of the measured electrical resistivity, respectively, and n denotes the number of the measured electrical resistivity.

## 3. Data Collection

### 3.1. Site Description and Measurement

**Figure 1:**
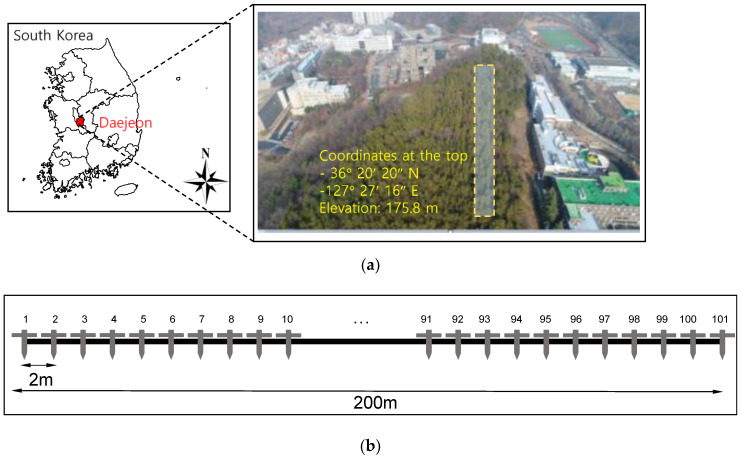
Site description: (**a**) research area; (**b**) electrode installation.

The experimental area of this study was a mountain with a history of landslides, located in Daejeon Metropolitan City, South Korea, and reinforcement techniques, including anchor and bolt, used for retaining walls and rockfall prevention networks were applied. The objective area was located near the downtown area, as depicted in Figure 1; hence, significant damage may be accompanied by landslides. The objective area was continuously monitored after the reinforcement technique was applied to analyze the behavior of the area and prevent landslide occurrence. The coordinates at the top of the mountain are 36° 20′ 20″ N and 127° 27′ 16″ E, and the elevation is approximately 175.8 m.

The straight line of the electrical resistivity was set to a length of 200 m at the mountaintop to cover all the reinforced areas. A Wenner array was selected because it is suitable for vertical measurements. The electrodes were installed at equal spacing intervals of 2 m, and a total of 101 electrodes were used. Stainless steel, which experienced a small oxidation reaction, was applied as the electrode material for long-term monitoring, and the length and diameter of the electrode were 0.5 and 0.01 m, respectively. Sting R1, purchased from AGI, was utilized to obtain the electrical resistivity. Voltage and current values of 10 V and 250 mV, respectively, were applied.

In this study, the electrical resistivity was measured for 15 months, from January 2019 to April 2020. Even though the values were measured on the 1st day and 15th day of each month, the electrical resistivity was also recorded in particular cases, including rainfall and temperature change. The number of measured data points at a given time was 1650, and a total of 49,500 (1650 × 15 months × 2 times) measurements were performed.

**Figure 2:**
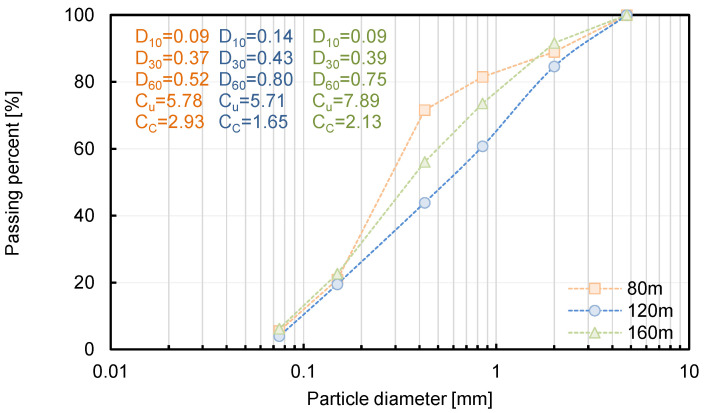
Results of sieve analysis. The D_10_, D_30_, and D_60_ denote the particle diameter corresponding to 10 [%], 30 [%], and 60 [%] passing percent. The coefficients of uniformity (C_u_) and curvature (C_c_) can be calculated for D_60_/D_10_ and D_30_^2^/D_60_D_100_, respectively.

The soil samples were collected at positions of 80 m, 120 m, and 160 m in the horizontal direction, and sieve analysis was performed to determine the characterization of the slope. The fine contents of 80 m, 120 m, and 160 m were calculated to be 3.9%, 3.9%, and 3.8%, respectively, as shown in Figure 2. The coefficients of uniformity (C_u_) were estimated to 5.78, 5.71, and 7.89 for each position, and the coefficients of curvature (C_c_) of 2.93, 1.65, 2.13 were deduced at positions of 80 m, 120 m, and 160 m, respectively. Finally, the study area was distributed with poor-graded sand (SP) through the unified soil classification system (USCS).

### 3.2. Distribution of Electrical Resistivity

**Figure 3:**
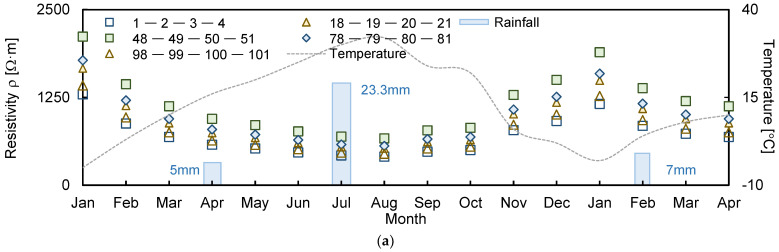

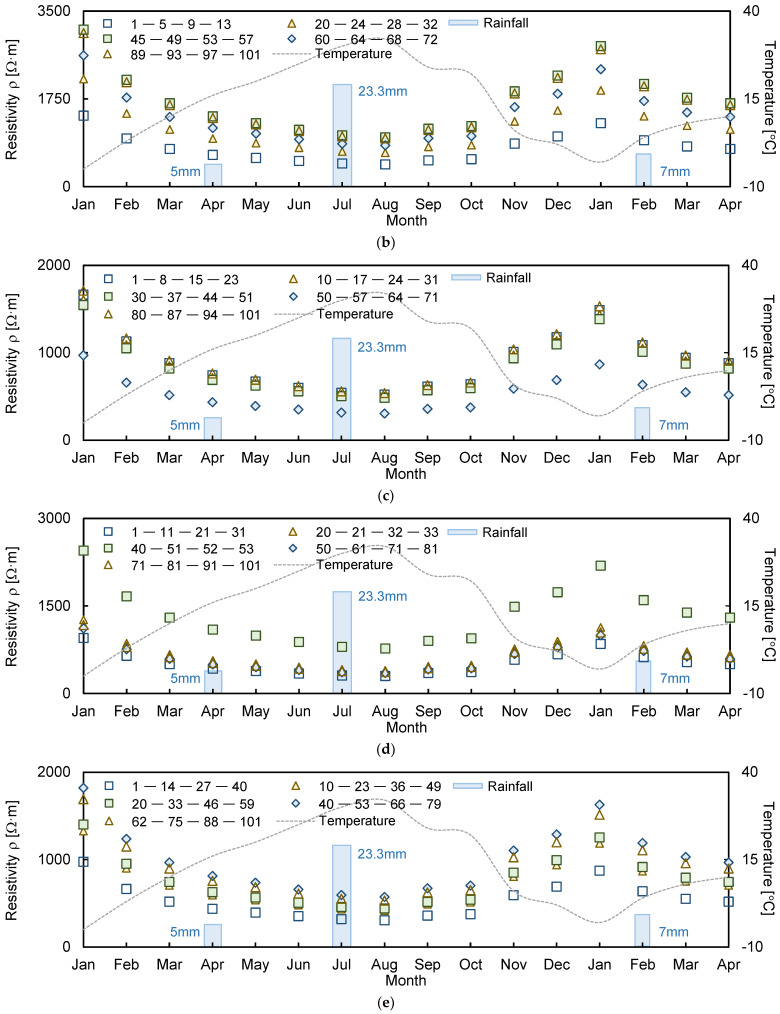

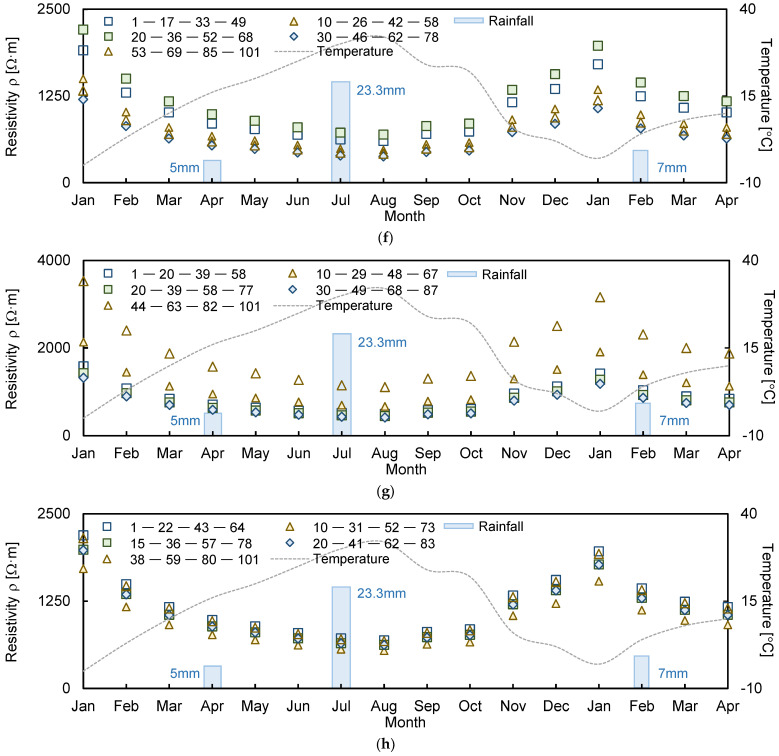
Distribution of measured electrical resistivity using electrode spacings of: (**a**) 1; (**b**) 4; (**c**) 7; (**d**) 10; **(e**) 13; (**f**) 16; (**g**) 19; (**h**) 21.

The electrical resistivity values measured over 15 months using each of the four electrodes are displayed in Figure 3, which indicates that the distributions of electrical resistivity were based on electrode spacings of 1, 4, 7, 10, 13, 16, 19, and 21. The electrodes were installed at 2 m intervals. For example, electrode spacings of 1 and 21 denote 2 and 42 m intervals of the electrodes, respectively. Temperature and rainfall values were also recorded and plotted, as shown in Figure 3.

The lowest and highest temperatures were observed in January and August, with values of −5 and 32 °C, respectively. Therefore, the electrical resistivity values measured in January and August were compared with the values obtained during March at 10 °C to assess the reliability of the measured values. The electrical resistivity for January was high, with ratios of 15.59%, 8.82%, 5.55%, 5.36%, 5.32%, 4.91%, 4.87%, and 3.93% for electrode spacings of 1, 4, 7, 10, 13, 16, 19, and 21, respectively. However, the measured values for August were relatively lower, with ratios of 31.12%, 24.32%, 23.62%, 21.84%, 21.80%, 21.31%, 14.78%, and 6.25% with an increase in electrode spacing. The temperature variation was related to the ionic activity inside the electrode material. When the temperature rises, the ions become more active, and the electrical conductivity increases [31]. Electrical resistivity, which is inversely proportional to electrical conductivity, decreases as the temperature increases, and this behavior was considered for determining the reliable electrical resistivity through the compensated method.

During the measurement period, 5 and 23.3 mm of rainfall fell in April and July of 2019, respectively. The electrical resistivity results obtained in March and June, which were the respective preceding months of rainfall, were compared to analyze the electrical resistivity behavior with respect to the rainfall. When the rainfall was 5 mm (April), the electrical resistivity decreased to 34.17%, 31.93%, 27.96%, 24.26%, 23.22%, 22.99%, 21.10%, and 9.17% for electrode spacings of 1, 4, 7, 10, 13, 16, 19, and 21, respectively. The electrical resistivity measured in July also decreased to 35.48%, 34.20%, 30.43%, 26.71%, 25.71%, 23.70%, 21.18%, and 17.96% with an increase in electrode spacing. This behavior is similar to that shown in previous research findings, in which the electrical resistivity decreased with an increase in ground moisture [32]. Furthermore, the electrical resistivity varied slowly with temperature and rainfall when the electrode spacing was increased because the deeper the depth, the slower the reaction to external environmental changes.

**Figure 4:**
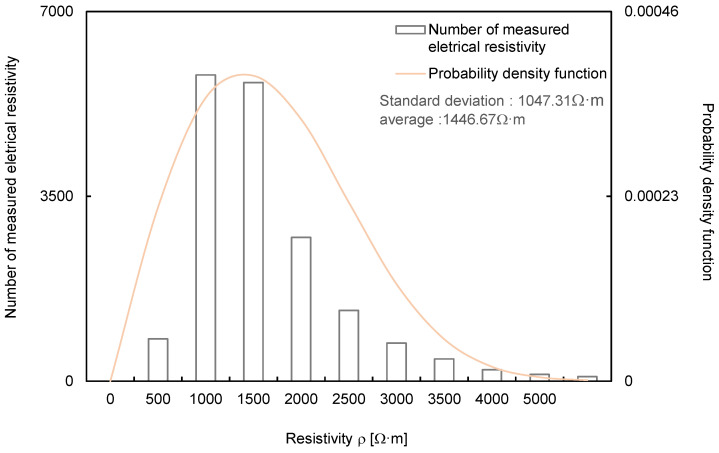
Normal distribution curve of the measured electrical resistivity.

The measured electrical resistivity values ranged from 1 to 3700 Ω·m at different locations and depths. The distribution curve of electrical resistivity is presented in Figure 4. The average value of the measured electrical resistivity was 1446.67 Ω·m, and the standard deviation was 1047.31 Ω·m. The reliability of the deep learning algorithms was compared using the reliable electrical resistivity values measured over the 16 months.

## 4. Application of the Deep Learning Algorithm

The deep learning procedure consisted of the normalization process, building model, and validation. The normalization process was necessary to increase the operation speed. The min–max scaling method was also used in this study. The measured electrical resistivity was converted to a range within 0 to 1 using Equation (13):

(13) $$\mathrm{MinMax}=\frac{\mathrm{ER}-{\mathrm{ER}}_{\min}}{{\mathrm{ER}}_{\max}-{\mathrm{ER}}_{\min}}$$

where ER denotes the corresponding input electrical resistivity, and ER_max_ and ER_min_ denote the maximum and minimum values of the measured electrical resistivity.

During the model building stage, the DNN, LSTM, GRU, LSTM-DNN, and GRU-DNN deep learning algorithms were selected, and the activation function, initialization method, number of hidden layers, number of nodes, optimization technique, iterative method, and sequence length were set. Six commonly used activation functions—hyperbolic tangent (tanh), sigmoid, softplus, rectified linear unit (relu), exponential linear unit (elu), and scaled exponential linear unit (selu) [33,34] were selected to compare the reliability of the algorithms. The initialization method was classified based on the applied activation function. In the cases of the tanh, sigmoid, and softplus functions, the Xaviera normal initialization method was used [35], while the He initialization method was applied to the relu, elu, and selu functions [36]. In this study, an appropriate initial weighting method was adopted according to the six selected activation functions. The number of hidden layers was set to 2, 10, and 20 layers in each of the algorithms. The numbers of nodes in the DNN algorithm were 20, 30, and 40, and the sequence lengths of LSTM and GRU were set to 1, 10, and 20. In addition, the number of nodes and the sequence lengths of the LSTM-DNN and GRU-DNN algorithms were identically configured based on the individual algorithm conditions. The optimization technique is an important method used for determining the weight and bias of each algorithm. The Adam optimizer, which combines the advantages of Adagrad [37] and RMSProp [38], was used. Finally, the early stopping technique was used as the iterative method to prevent overfitting.

**Figure 5:**
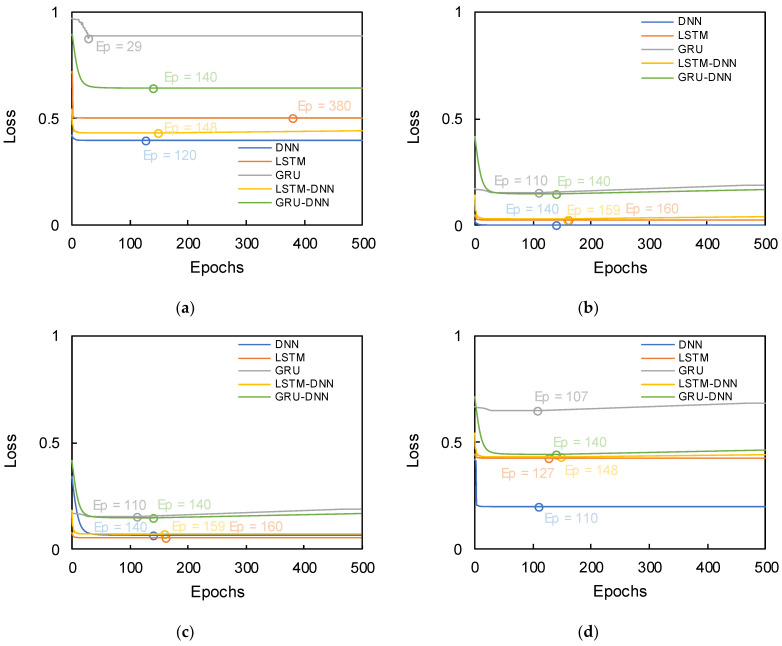
Results of loss based on ratio of training, validation and test data: (**a**) 7:2:1; (**b**) 6:2:2; (**c**) 5:2:3; (**d**) 4:2:4. The hollow circles denote the optimal epochs.

Validation is necessary to examine the reliability of the hyperparameters set in each algorithm [39], and the hold-out validation approach, which is typically applied, was also selected in this study. To find the best ratio of validation, the results of loss were plotted in Figure 5 with ratios of (7:2:1), (6:2:2), (5:2:3), and (4:2:4) for training, validation, and test data, respectively. Figure 5 shows that the reasonable trend was distributed without overfitting in selected ratios, and the optimal epoch was automatically selected through the early stopping technique. The best epoch and loss were summarized in Table 1. The ratio of 6:2:2 demonstrated the lowest loss in the range of 0.0001 to 0.145, and thus, the ratio was adopted to increase resolution in this study. To perform the deep learning algorithm, the Keras (version 2.2.4) package was used with Python language (version 3.7.4).

## 5. Results

**Figure 6:**
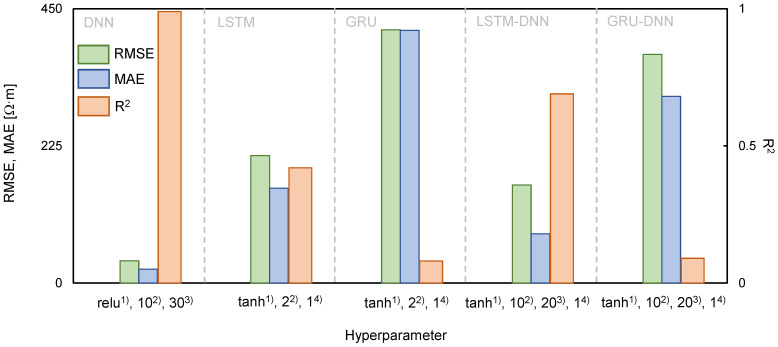
Comparisons of The root mean square error (RMSE), mean absolute error (MAE), and coefficient of determination (R^2^) based on best hyperparameter. The ^1)^, ^2)^, ^3)^, and ^4)^ denote activation function, the number of hidden layers, number of nodes, and sequence length, respectively.

**Figure 7:**
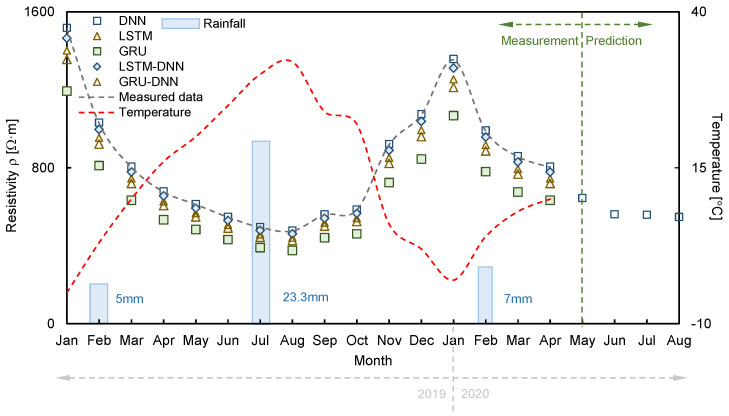
Pattern analysis by comparison of measured and predicted electrical resistivity.

**Figure 8:**
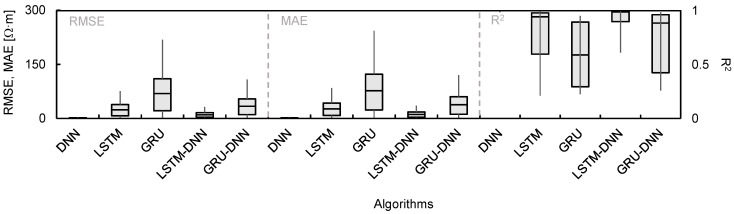
Box plots of DNN, LSTM, GRU, LSTM-DNN, and GRU-DNN between the measured and predicted electrical resistivity.

The RMSE, MAE, and R^2^ were calculated using Equations (10)–(12) to evaluate the reliability of the hyperparameters set in each algorithm. A reverse min–max scaling method was applied to obtain the electrical resistivity because the predicted values using the deep learning algorithms were normalized. In Figure 6, each hyperparameter was divided into groups depending on the deep learning algorithms for DNN (activation function, number of hidden layers, and number of nodes) and both LSTM and GRU (activation function, number of hidden layers, and sequence length). LSTM-DNN and GRU-DNN were combined with the activation function, number of hidden layers, number of nodes, and sequence length for comparison with the calculated error. DNN, LSTM, and GRU were marked as 56 types, and LSTM-DNN and GRU-DNN were classified as 162 cases. The calculated RMSE, MAE, and R^2^ are summarized in Table 2. The hyperparameters in which the RMSE and MAE values were minimum and R^2^ was maximum were DNN (Relu, 10, 30), LSTM (tanh, 2, 1), GRU (tanh, 2, 1), LSTM-DNN (tanh, 10, 20, 1), and GRU-DNN (tanh, 10, 20, 1). These conditions were considered the most reliable hyperparameters and are listed in Table 3. The RMSE, MAE, and R^2^ were evaluated using DNN (36.38 Ω·m, 22.62 Ω·m, 0.99), LSTM (209.12 Ω·m, 155.47 Ω·m, 0.42), GRU (415.56 Ω·m, 414.59 Ω·m, 0.08), LSTM-DNN (160.71 Ω·m, 80.98 Ω·m, 0.69), and GRU-DNN (375.25 Ω·m, 306.05 Ω·m, 0.09), as shown in Figure 6. Hence, the order of the optimal deep learning algorithms for predicting electrical resistivity under these experimental conditions was found to be DNN > LSTM-DNN > LSTM > GRU-DNN > GRU. However, even though LSTM-DNN was the second most reliable algorithm after DNN, the difference in the error values between these two algorithms was large. It should be noted that DNN adequately reflected the trend in the measurement data. A pattern analysis of electrical resistivity was conducted by comparing the measured values with the predicted values based on the hyperparameters with high reliability, and the results of each deep learning algorithm are displayed in Figure 7. The 3300 data measured each month were averaged and plotted to represent each month. Among the deep learning algorithms, DNN showed the most similar trends and values to the measured data (Figure 7). However, the values predicted using the other algorithms had a low electrical resistivity range, which resulted in insufficient reliability. Discrepancies were calculated using Equations (10)–(12) to quantitatively analyze the accuracy of each algorithm, as shown in Figure 8. The averaged RMSE, MAE, and R^2^ groupings of the DNN, LSTM, GRU, LSTM-DNN, and GRU-DNN algorithms were (2.96 Ω·m, 3.29 Ω·m, 0.98), (60.82 Ω·m, 67.58 Ω·m, 0.77), (175.33 Ω·m, 194.81 Ω·m, 0.59), (25.80 Ω·m, 28.66 Ω·m, 0.92), and (86.38 Ω·m, 95.98 Ω·m, 0.73), respectively. DNN exhibited greater reliability than the other deep learning algorithms based on quantitative values. The DNN algorithm clearly enhances the reliable prediction of electrical resistivity by reflecting the changes in external environmental conditions, including rainfall and temperature.

## 6. Discussion

### 6.1. Prediction of Porosity

**Figure 9:**
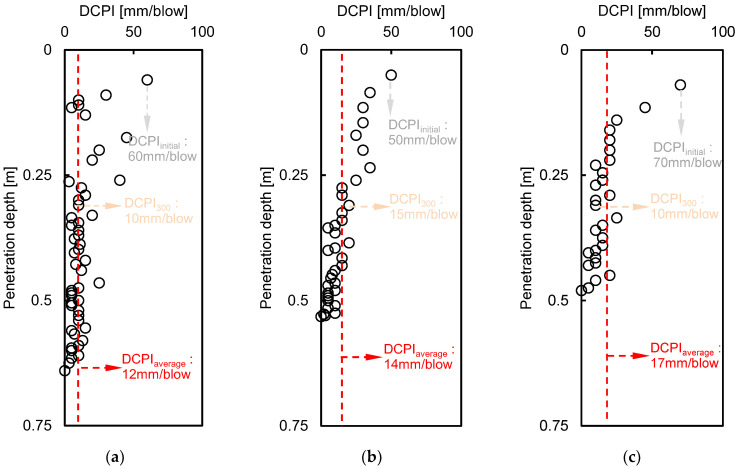
Results of dynamic cone penetrometer index (DCPI) through a dynamic cone penetrometer test (DCPT) at horizontal positions of (**a**) 80 m; (**b**) 120 m; (**c**) 160 m.

According to the results of the sieve analysis, the fine content was less than 4.2%, and the area was almost entirely composed of sand particles based on the unified soil classification system (USCS). Therefore, the intrinsic formation factor of Equation (2) can be applied as the apparent formation factor, and the measured value was used for the bulk electrical resistivity. The alternation of fluid electrical resistivity was assumed to be small at each position because the change of ions in a porous medium is tiny due to the small content of the fine particles. Moreover, a constant fluid electrical resistivity of 116.27 Ω·m, measured in the stored rainfall through the 4-electrode technique, was used. More information on the 4-electrode technique can be found in Taiwo et al. [30]. The estimated porosity based on a dynamic cone penetrometer test (DCPT) was estimated to obtain the tortuosity factor and the cementation factor through inversion. The results of DCPT at a horizontal distance of 80 m, 120 m, and 160 m are shown in Figure 9.

**Figure 10:**
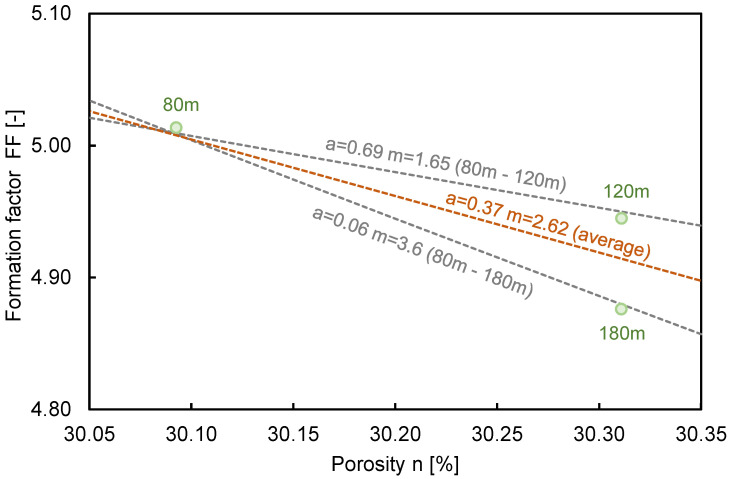
Relationship between the formation factor and porosity for the extracted specimens. Here, a and m denote the tortuosity and cementation factors.

**Figure 11:**
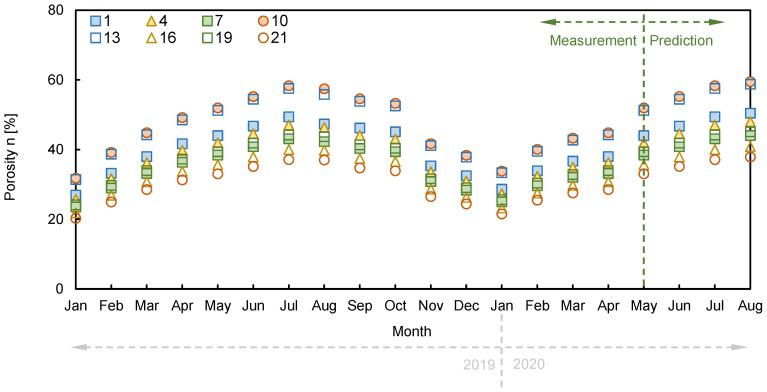
Distribution of porosity according to a time series.

The DCPI, which provides the penetration depth per blow, revealed about a 50–70 mm/blow around the surface, with close to 0 mm/blow at the bottom of the depth. The final penetration depth of DCPT was recorded as 0.64 m, 0.53 m, and 0.48 m at distances of 80 m, 120 m, and 160 m, respectively, due to spatial distribution. The porosity was determined to be 30.09%, 30.11%, and 30.11% at distances of 80 m, 120 m, and 160 m, respectively, using the DCPI recorded at the surface and Equation (3). The DCPI_300_ was estimated as 10, 15, and 10 mm/blow at positions of 80 m, 120 m, and 160 m, respectively, and the D_50_ was calculated as 0.48, 0.67, and 0.55 at each horizontal distance through a sieve test, as shown in Figure 2. The measured electrical resistivity values at 80 m, 120 m, and 160 m were 573 Ω·m, 585 Ω·m, and 567 Ω·m, respectively, in February 2020, when the DCPI experiment was conducted, and the tortuosity factor and cementation factor were calculated through simultaneous equations with a fluid electrical resistivity of 116.27 Ω·m. The ranges of the tortuosity factor and cementation factor based on 80 m and 120 m, 80 m, and 160 m were 0.01-0.69 and 1.65-3.6, as shown in Figure 10, and the averaged tortuosity factor and cementation factor of 0.37 and 2.62 were used. The distributions of the calculated porosity based on measured electrical resistivity are plotted in Figure 11. The electrode spacing was selected as 1, 4, 7, 10, 13, 16, 19, and 21, with the same value of Figure 3, and the ranges of the calculated porosity based on the measured and predicted electrical resistivities were 20–59% and 33–59%, respectively. The highest porosity was shown in July when rainfall occurred. Equation (2) shows that the porosity was dependent on the electrical resistivity when the constant values were fixed.

Thus, the distributions of porosity showed similar behavior to the trend of electrical resistivity. Although the types of pores, specific surface areas, tortuosity, and overburden pressure may change when rainfall or external conditions occur, there is a limit to evaluating the detailed behaviors reflecting seasonal variation. Therefore, porosity distributions were presented, focusing on the overall behavior.

### 6.2. Prediction of Hydraulic Conductivity

**Figure 12:**
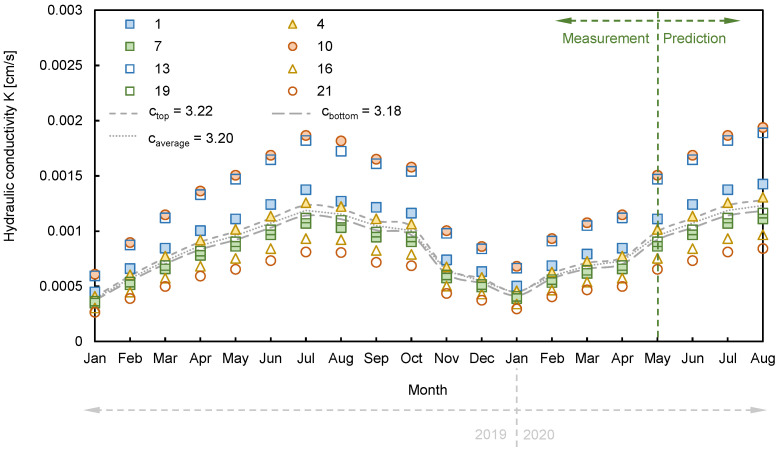
Distribution of hydraulic conductivity according to a time series.

Hydraulic conductivity was converted using the measured electrical resistivity and Equation (4). For the shape factor, an average value of 8.4 was used [40], and the average effective diameter of 0.59 mm was applied, with reference to the result of the sieve analysis, as shown in Figure 2. Thus, the specific surface area per unit volume (S_p_), which is the ratio between the shape factor and the effective diameter, was calculated as 14.23 (mm^−1^). The value of c was derived as another constant by inverting the measured hydraulic conductivity from the constant head test. The specimens were extracted at the top and bottom of the slope, and the hydraulic conductivity levels were measured as 0.0005 cm/s and 0.00049 cm/s, respectively. Finally, the c values were calculated as 3.22 and 3.18 at the top and bottom of the slope, and an average value of 3.2 was used to find the distributions of hydraulic conductivity, as shown in Figure 12. From January 2019 to April 2020, which was the range of measured electrical resistivity, hydraulic conductivity was repeatedly increased and decreased by about 0.00027–0.00194 cm/s. In addition, hydraulic conductivity tended to increase slightly after May 2020, and the predicted hydraulic conductivities were observed in the range from 0.00045 cm/s to 0.00143 cm/s. The hydraulic conductivity also had a strong influence on electrical resistivity when the constant value was fixed. Thus, the distribution of hydraulic conductivity was similar to the trend of electrical resistivity. Although it was difficult to capture the detailed input constant according to monthly strata changes, it was possible to determine the overall behavior of hydraulic conductivity.

### 6.3. Discontinuity Depth

**Figure 13:**
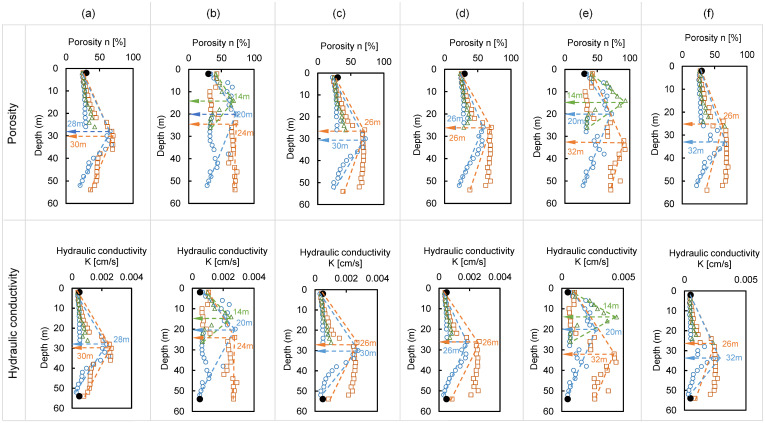
Discontinuity depth assessment based on porosity and hydraulic conductivity: (**a**) February 2019; (**b**) July 2019; (**c**) May 2020; (**d**) June 2020; (**e**) July 2020; (**f**) August 2020. The blue circles, red rectangles, and green triangles denote 80 m, 120 m, and 160 m horizontal distance, respectively, and each symbol is connected with a dotted straight line. The solid circles with black color indicate the measured values.

Landslides often occur at the discontinuous surface of a stratum and the interface between unsaturated and saturated soils [41]. According to Vieira and Fernandes [42], an area with a highly different ratio of gravel–sand–silt–clay is generally considered dangerous and can be inferred by changes in design parameters, including porosity and hydraulic conductivity. In this study, the porosity and hydraulic conductivity in the horizontal directions of 80 m, 120 m, and 160 m, which indicate a large change in electrical resistivity, are addressed in Figure 13 for discontinuity depth with a maximum depth of 52 m, 54 m, and 26 m, respectively considering skin depth of electrical resistivity. The data were divided into the dry season (February 2019) and wet season (July 2019), and the range of porosity and hydraulic conductivity in the dry season was 20.97–70.22% and 0.00028–0.002631 cm/s, respectively. During the rainy season, the range of porosity and hydraulic conductivity was 28.12–73.22% and 0.00049–0.00289 cm/s, and the change ratios were calculated to be up to 34.08% and 71.82% for porosity and hydraulic conductivity. The predicted porosity and hydraulic conductivity after May 2020 are also shown, and the ranges of the porosity and hydraulic conductivity for May, June, July, and August 2020 were 22.26–70.39%, 0.00031–0.0028 cm/s; 22.17–70.39%, 0.00031–0.0026 cm/s; 28.12–93.22%, 0.000486–0.0044 cm/s; and 22.24–70.91%, 0.0031–0.0027 cm/s. The porosity and hydraulic conductivity in July 2020, when rainfall was predicted, increased by up to 32.43% and 67.94%, respectively, compared to dry (May, June, and August 2020), which was similar to the measurement results for 2019. The measured porosity and hydraulic conductivity through DCP and constant head test at a depth of 2 m and 50 m, respectively, are also demonstrated in Figure 13 with a solid circle, and they show similarity with estimated values through electrical resistivity. Even though the additional tests for obtaining porosity and hydraulic conductivity were performed in February 2019, the measured values are totally described in Figure 13a–f for efficiently comparing values. The tendency of porosity and hydraulic conductivity gradually increased with depth. However, the opposite trend was observed under average depths of about 29 m (February 2019), 19 m (July 2019), 28 m (May 2020), 26 m (June 2020), 22 m (July 2020), and 29 m (August 2020), as shown in Figure 13. However, it was difficult to find the opposite trend at a distance of 160 m because the skin depth was only 26 m. The estimated depths, in detail, at each position of 80 m, 120 m, and 160 m are addressed in Table 4. The bilinear depth showed discontinuity of the porosity and hydraulic conductivity. In the dry season, the average depth was estimated to be 29 m. However, the depth was 19 m on average in the rainy season. The difference in the discontinuity depth under dry and wet conditions was about 10 m based on measured data. The predicted data also demonstrated a similar trend, and the different discontinuous depth of dry and rainy seasons was calculated to 6–7 m. Thus, caution is needed to find discontinuous areas depending on external conditions. These results show that discontinuity depths can be predicted through deep learning algorithms, and it is expected that more precise predictions could be performed with a larger amount of data.

## 7. Conclusions

Deep learning algorithms have been used in various fields to predict the future behaviors of objects. In this study, the DNN, LSTM, GRU, LSTM-DNN, and GRU-DNN deep learning algorithms were applied to predict electrical resistivity, which indicates the behaviors of geotechnical engineering. The detailed conclusions of this study are as follows:

- Electrical resistivity was measured on a mountaintop over 15 months, and the number of accumulated data points was 49,500. The measured electrical resistivity reflected changes in rainfall and temperature.
- DNN, which is generally used as an algorithm in neural networks, was selected for predicting electrical resistivity. Additionally, LSTM and GRU, which are based on the RNN algorithm, were used because they can reflect past and present conditions as a time series. LSTM-DNN and GRU-DNN, which are complex algorithms, were also used to improve the reliability.
- The electrical resistivity prediction indicated excellent performance in the following order: DNN > LSTM-DNN > LSTM > GRU-DNN > GRU.
- Porosity and hydraulic conductivity were predicted through electrical resistivity, and the discontinuity depth was also estimated by porosity and hydraulic conductivity.

## Figures and Tables

**Table 1:** Distribution of best epochs with loss based on the ratio of training, validation, and test data. The DNN, LSTM and GRU denote deep neural network, long-short term memory, gated recurrent unit, respectively. The LSTM-DNN and GRU-DNN show coupled algorithms based on DNN with LSTM and GRU.

Ratio	7:2:1		6:2:2		5:2:3		4:2:4	
Algorithms	Epochs	Loss	Epochs	Loss	Epochs	Loss	Epochs	Loss
DNN	120	0.40	140	0.0001	140	0.65	110	0.20
LSTM	380	0.50	160	0.025	160	0.05	127	0.42
GRU	29	0.88	110	0.15	110	0.15	107	0.65
*LSTM-DNN*	148	0.43	159	0.025	159	0.07	148	0.43
*GRU-DNN*	140	0.64	140	0.145	140	0.14	140	0.44

**Table 2:** Root mean square error (RMSE), mean absolute error (MAE), and coefficient of determination (R^2^) based on various hyperparameters.

Validation	DNN	LSTM	GRU	LSTM-DNN	GRU-DNN
RMSE	36.38–498.56 [Ω·m]	209.12–498.56 [Ω·m]	415.56–798.56 [Ω·m]	160.72–882.86 [Ω·m]	375.25–782.86 [Ω·m]
MAE	22.62–445.32 [Ω·m]	155.47–441.00 [Ω·m]	414.59–740.98 [Ω·m]	80.98–845.00 [Ω·m]	306.05–775.46 [Ω·m]
R^2^	0.00–0.99	0.01–0.42	0.00–0.08	0.01–0.69	0.01–0.09

**Table 3:** Reliable hyperparameters of deep neural network (DNN), long-short term memory (LSTM), and gated recurrent unit (GRU), LSTM-DNN, and GRU-DNN.

Hyperparameter	DNN	LSTM	GRU	LSTM-DNN	GRU-DNN
Activation function	Relu	tanh	tanh	tanh	tanh
Initialization method	He Initialization	Xavier Normal Initialization	Xavier Normal Initialization	Xavier Normal Initialization	Xavier Normal Initialization
Number of hidden layers	10	2	2	10	10
Number of Node	30	-	-	20	20
Sequence length	-	1	1	1	1
Optimizer technique	Adam	Adam	Adam	Adam	Adam

**Table 4:** Estimated discontinuity depth based on the discontinuity of porosity and hydraulic conductivity.

	Measurement Depth (2019)		Predicted Depth (2020)			
Horizontal Distance	February	July	May	June	July	August
80 [m]	28 [m]	20 [m]	30 [m]	26 [m]	20 [m]	32 [m]
120 [m]	30 [m]	24 [m]	26 [m]	26 [m]	30 [m]	26 [m]
160 [m]	-	14 [m]	-	-	14 [m]	-
Averaged value	29 [m]	19 [m]	28 [m]	26 [m]	22 [m]	29 [m]
